# Antenatal Corticosteroids: A Risk Factor for the Development of Chronic Disease

**DOI:** 10.1155/2012/930591

**Published:** 2012-03-05

**Authors:** Elizabeth Asztalos

**Affiliations:** Department of Paediatrics, University of Toronto and The Centre for Mother, Infant, and Child Research, Sunnybrook Research Institute, Sunnybrook Health Sciences Centre, 2075 Bayview Avenue, Toronto, ON, Canada M4N 3M5

## Abstract

Preterm birth remains a major health issue worldwide. Since the 1990s, women at risk for preterm birth received a single course of exogenous antenatal corticosteroids (ACSs) to facilitate fetal lung maturity. More recently, repeated or multiple courses of ACS have been supported to provide continued fetal maturity support for women with continued risk of preterm birth. However, exogenous ACS reduces birth weight which, in turn, is associated with adverse adult outcomes such as coronary heart disease, stroke, hypertension, and type 2 diabetes. The long-term effects of ACS exposure on HPA axis activity and neurological function are well documented in animal studies, and it appears that ACS, regardless of dose exposure, is capable of affecting fetal HPA axis development causing permanent changes in the HPA axis that persists through life and is manifested by chronic illness and behavioral changes. The challenge in human studies is to demonstrate whether an intervention such as ACS administration in pregnancy contributes to developmental programming and how this is manifested in later life.

## 1. Introduction

The “developmental origins of health and disease” (DOHaD) refers to a concept where exposure to environmental factors such as maternal nutrition, body composition, and stress hormone levels sends signals to a developing fetus that potentially influences the metabolic phenotype of the offspring and its risk of chronic diseases later in life [1–5]. This concept was originally developed to explain observations between the high rates of death due to coronary heart disease in areas of England and Wales with high neonatal mortality and proposed that intrauterine deprivation was an important mediator [6]. Further studies found an inverse relationship between birthweight and coronary heart disease [7]. The association of low birth weight and increased incidence of chronic illnesses of adulthood, such as heart disease, hypertension, type 2 diabetes, has been documented in numerous studies [8–12]. Low birth weight has been proposed as an indicator of an environmental adversity during fetal development and may suggest fetal processes or programming are in place. Two mechanistic hypotheses have been proposed to explain how fetal programming may arise: fetal malnutrition and fetal overexposure to glucocorticoids both of which may have effects either directly or indirectly upon the developing fetus. Exposure to excess glucocorticoids in utero acts to program the fetal hypothalamic-pituitary-adrenal (HPA) axis, permanently altering basal and stress-induced HPA axis activity and regulation in the offspring throughout life. Fetal exposure to excess glucocorticoids can occur via a number of mechanisms such as maternal stress during pregnancy and maternal treatment with synthetic glucocorticoids (sGC), more commonly known as antenatal corticosteroids (ACS). 

This paper will focus on ACS and its effect on the developing HPA axis and building the case for the use of ACS as a risk factor for the development of chronic disease in the offspring.

## 2. Development and Function of the Hypothalamic-Pituitary-Adrenal (HPA) Axis

Activation of the HPA axis after exposure to a stressor is part of a normal adaptive response allowing the organism to respond to changes in its environment [13]. Stress results in activation of central neurocircuitry, which in turn signals the hypothalamus to produce and secrete corticotrophin-releasing hormone (CRH) and vasopressin (AVP). CRH and AVP lead to the release of adrenocorticotrophic hormone (ACTH) from the anterior pituitary which stimulates the production and secretion of glucocorticoids (GC), principally cortisol in humans. Cortisol binds the glucocorticoid receptors (GR) in multiple target tissues to maintain homeostasis following stress. Cortisol also binds to GR and mineralocorticoid receptors (MR) in the limbic regions (including hippocampus and prefrontal cortex) to modify behaviours and learning and memory. In addition, cortisol acts to feedback and decrease HPA function in a classic endocrine feedback loop. Tightly regulated feedback control of cortisol is critical as prolonged tissue exposure to GC leads to metabolic and behavioural disorders [14]. The timing of maturation of the HPA axis relative to birth is highly species-specific and is linked to landmarks of brain development. In humans, like animals that give birth to mature young (primates, sheep, and guinea pigs), maximal brain growth and a large proportion of neuroendocrine maturation takes place *in utero* [15, 16].

## 3. Role of 11***β***-HSD-2

Low levels of GC are critical for normal fetal development; however, fetal exposure to elevated glucocorticoids can inhibit growth and adversely impact brain development. Under normal conditions, access of maternal cortisol to the fetus is low due to the placental expression of the enzyme, 11*β*-hydroxysteroid dehydrogenase-2 (11*β*-HSD-2) [17]. Placental 11*β*-HSD-2 transforms cortisol into its much less active 11-keto form, cortisone. This protective placental enzymatic barrier is very efficient, such that only 10–20% of maternal cortisol crosses the placenta and reaches the fetus. This 10–20% passage of active maternal GC to the fetus reflects an anatomical bypass of the enzyme, presumably adding to the provision of GC for normal key developmental processes in the fetuses such as maturation of the lung and other fetal organs [18].

Low levels of placental 11*β*-HSD-2 activity have been correlated with low birth weight in humans and rodent models [19–22]. Reduced levels of 11*β*-HSD-2 may contribute to greater signaling within the placenta itself which may impact fetal development by altering placental function. Rodent studies have demonstrated that variations in placental 11*β*-HSD-2 levels have correlated with altered expression in glucose and amino acid transporter molecules as well as growth factors in the placenta [23].

It is clear that placental 11*β*-HSD-2 plays a key role in mediating the flow of environmental signals to the fetus. Animal studies have demonstrated that prenatal stress can lead to a reduction in placental 11*β*-HSD-2 activity suggesting then that the fetus and placenta are exposed to extra excessive amounts of GC [24]. In addition, dietary protein restriction selectively reduces 11*β*-HSD-2 activity suggesting a common mechanism by which malnutrition and excess GC can programme adult biology [25, 26].

Unlike endogenous GC, the synthetic GCs utilized as ACS (dexamethasone and betamethasone) are poor substrates for 11*β*-HSD-2 and readily passes the placenta barrier. This lack of restriction allows ACS to exert its effect in the same manner as excessive endogenous GC.

## 4. Other Stress-Related Programming Mediators

While it is clearly evident the role GC has on influencing development under normal and stress-related circumstances, other substances are released by the mother and fetus in response to stress. Catecholamines, in particular adrenaline and noradrenaline, are of importance since they are both released by stress and may influence placental function and also transport across the placenta to the fetus [27]. However, more exploration in this area is required to determine how these catecholamines function in normal and abnormal situation and what contribution they may play in enhancing disease risk.

## 5. Use of Antenatal Corticosteroids (ACSs) in the Management of Women at Risk for Preterm Birth

Preterm delivery affects over 7–12% of births in North America alone and is responsible for up to 75% of neonatal deaths [28, 29]. Despite advances in medical technology, the prevalence of preterm birth worldwide is increasing [29].

Respiratory distress syndrome, as a consequence of immature lung development, is a significant risk of preterm birth and the major cause of early neonatal mortality and morbidity [30]. Infants born very preterm (less than 32 weeks of gestation) often require respiratory support, in the form of positive pressure ventilation and prolonged oxygen support. A substantial proportion have intraventricular haemorrhages and associated “white matter brain injury” (grade 3 or 4 intraventricular haemorrhages, periventricular leukomalacia), bronchopulmonary dysplasia, and severe retinopathy of prematurity, all of which contribute significantly independently and combined to an adverse neurodevelopmental outcome in later life [31, 32]. Infants born preterm who survive have an increased risk of hospitalizations [33]. The personal and emotional costs for affected individuals and their families are high, as are the immediate and long-term monetary costs of these morbidities for parents and society [33]. Thus, preterm birth and preterm infants continue to remain a significant health issue worldwide.

The immature fetal lung is the target organ when women at risk of preterm birth are given ACS. Stimulation of the pulmonary surfactant system has been regarded as the most important effect of ACS. In addition, ACS can alter lung fluid absorption and alveolar development by inducing genes associated with the synthesis of surfactant proteins, fatty acid syntheses, the epithelial sodium channel, and the membrane protein sodium/potassium ATPase [34, 35]. The enhancement of these mechanisms causes an accelerated maturation of the fetal lung and reduces the severity of respiratory distress syndrome (RDS) in the first few days after a preterm birth. This, in turn, contributes to a reduction in mortality and other neonatal morbidities associated with preterm birth. In 1972, Liggins and Howie published the results of the first RCT evaluating the effects of a single course of ACS [36]. Among those women who had been in spontaneous preterm labour, ACS reduced the risk of respiratory distress syndrome (RDS) (9.0% versus 25.8%, *P* = 0.003) and early neonatal mortality (3.2% versus 15.0%, *P* = 0.01). Over the subsequent 20 years, 12 additional RCT, involving over 3000 infants, showed a benefit of a single course of ACS with no adverse effects [37].

Since the early 1990s, the recommendation has been that women, between 24 and 34 weeks of gestation and at high risk of preterm birth, receive a single course of ACS (2 doses of ACS 12 hours apart) [38, 39]. However, approximately 50% of women given a first course of ACS remain undelivered 7–14 days later, and, for these women, the question had arisen as to whether repeated courses of ACS should be given [40]. There has been no consensus on repeated courses of ACS in the management of women at risk for preterm birth [41]. In the past decade, 10 trials have been conducted to evaluate the risks and benefits of repeated courses of ACS to women at risk of preterm birth. The most recent review in the Cochrane Database of these trials involving over 4730 women and 5650 infants concludes that repeat courses of ACS does reduce the risk of RDS (risk ratio (RR) 0.83, 95% confidence interval (CI) 0.75–0.91), and serious infant outcomes (RR 0.84, 95% CI 0.75–0.94) compared to no repeat ACS treatment [42]. However, these benefits were associated with a significant reduction in size at birth. Because of this reduction in size at birth, concern over the long-term effects in the offspring from repeated courses of ACS has been raised, and more research on the long-term effects has been recommended [43].

## 6. Long-Term Clinical Significance of ACS Exposure in the Offspring

Dalziel et al. reported on the cohort in the initial trial by Liggins and did not identify overt neurological and physiological effects of a single course of ACS in late gestation but did identify early markers of insulin resistance [44, 45]. Retrospective studies evaluating the effect of repeated courses on neonates and children have been somewhat conflicting; while they suggest benefit in terms of lung function in the neonatal period, there appears to be an increased risk of other outcomes, namely, long-term effects on behavior [46–49]. The 2-year followup of the recent RCT evaluating the use of repeated courses of ACS has reported no significant differences in the rate of deaths or major neurodevelopmental difficulties at 2 years of age [42, 50–54]. However, caution must be exercised because measurements at 2 years of age have limited predictive abilities and are only moderately correlated with developmental outcomes at later ages [55]. In addition, these outcome studies did not address the question of potential programming effect from ACS and impact on chronic illness development. Several ongoing studies are currently taking place to evaluate the children closer to school age which include the Canadian Institutes of Health Research (CIHR)- funded Multiple Antenatal Corticosteroids Study 5-year followup (MACS-5) and the 6-year evaluation of the ACTORDS participants [56]. MACS-5 is specifically designed to evaluate the effect of ACS on the developing brain, specifically the hippocampus, by assessing memory, attention, and behaviour as well as neurocognitive abilities; the ACTORDS study has focused on neurocognition in addition to other developmental abilities as well as early biomarkers for chronic illness [56].

## 7. Potential Effects of ACS during Pregnancy

Much of what is known about the effect of ACS on various structures is from animal studies.

### 7.1. Brain Development

Endogenous GC is important for normal development of the central nervous system; circulating levels of GC are maintained at very low levels throughout the majority of gestation [57, 58]. However, levels will change to meet the needs of the developing brain during “critical windows.” Studies have shown that 11*β*-HSD-2 in the fetal human brain is silenced between 19 and 26 weeks which would lead to localized increases in GC signaling [59, 60]. GC is necessary for neuronal maturation, remodeling of axons and dendrite and cell survival [61, 62]. However, sustained elevation or depletion of GC during these “critical windows” of fetal development can modify brain structure with significant consequences to future function [63, 64]. Prenatal GC administration has been shown to retard brain weight at birth in sheep [65], as well as delay maturation of neurons, myelination, glia, and vasculature [60]. It has been shown to have widespread effect on neuronal structure and synapse formation and altered hippocampal structure and volume which can affect memory and attention [60, 65–68].

The hippocampus highly expresses GR and MR and, as such, is vulnerable to high sustained levels of GC. Rodent models show that prenatal stress decreases synaptic spine density and neurogenesis which correlates with deficits in cognition and learning. Similar effects are seen in primates (baboons and rhesus monkeys) where exposure to dexamethasone or betamethasone leads to reduced levels of neuritogenesis and neuronal plasticity and pronounced degeneration of areas within the hippocampus. These effects were dose associated suggesting multiple exposures induced more severe damage that a single injection [68–72].

### 7.2. HPA Axis

A number of animal models have shown that maternal administration of sGC during pregnancy has profound acute and long-term effects on the developing HPA axis. The fetal brain contains high levels of GR over the 2nd and 3rd trimesters, with highest levels in the limbic system (which includes prefrontal cortex, hippocampus and amygdala), the hypothalamus paraventricular nucleus (PVN; source of CRH and AVP), and the anterior pituitary (site of ACTH production) [73]. Exposure of these structures to high levels of GC can lead to permanent programming of the function of these structures. This “programming” takes place via modification of both the GR and MR levels in the hippocampus, hypothalamus, and pituitary gland. Prenatal exposure to sGC leads to permanent changes in the expression of GRs and MRs in these structures resulting in altered negative feedback sensitivity and altered set points for HPA function. Long-term changes in the regulation of HPA function can predispose to chronic cardiometabolic and neurological disorders. In addition, imbalance of GR and MR in the limbic system can have profound effects on behaviours, including learning and attention [74].

The effects of sGC are also noted to be sex dependent [75, 76]. Administration of a single course of sGC in a guinea pig model in a dose and regimen comparable to that used in humans demonstrated significantly increased levels of MR and GR in the brains of female-exposed guinea pigs. Multiple course exposure to sGC in the same model resulted in dose-dependent reductions in hippocampal NMDA-receptors in the hippocampus and modification of hippocampal long-term potentiation (LTP; the biological substrate of learning and memory), in female but not male offspring [77, 78].

Limited studies have been undertaken to establish the long-term effects of fetal exposure to sGC on the HPA axis function in nonhuman primates. Uno et al. [79] and de Vries et al. [80] were both able to demonstrate that repeated exposure to sGC in rhesus monkeys and Vervet monkeys resulted in offspring demonstrating elevated cortisol levels and altered responses to stress throughout life.

## 8. Mechanisms of Programming for ACS

A key aspect of the DOHaD hypothesis is that the effects of the environment are mediated by physiological and metabolic effects during fetal and early postnatal life [1–5]. The HPA axis is a central mediator of the programming process. Prenatal stress leads to numerous cardiovascular and endocrine changes in the mother, including increases in plasma ACTH, *β*-endorphin, cortisol, and catecholamine concentrations. Although the placenta acts as a barrier to many of these maternal factors, a number will still pass to the fetus because of potential attenuation of 11-*β*-HSD-2 in noncritical windows. There may be activation of the fetal sympathetic nervous system which will contribute to programming of the HPA axis and lead to an altered physiological response in the fetus. Administration of ACS can be seen as mimicking “*prenatal stress.*” Its effects are more direct as it is able to bypass the functional barrier of placental 11-*β*-HSD-2 and bind directly to the GR and MR in the vulnerable regions of the hippocampus and alter HPA axis activity. ACS has been shown from the clinical trials to affect in utero weight gain and lead to a reduced birth weight [42], a surrogate crude measure which has been associated with the development of hypertension, type 2 diabetes, and cardiovascular disease in adulthood [81].

### 8.1. Role of Epigenetics

There is evidence that the early environment can permanently influence the genome through epigenetic mechanisms and in this way modifies endocrine function, metabolism, and behaviour of offspring [82–87]. Gene expression can be epigenetically modified through alterations in DNA methylation and chromatin structure (i.e., histone acetylation). DNA methylation is a process where a methyl group is covalently linked to cytosine [88]. Methylation patterns are established during early embryonic development and maintained by DNA methyltransferases [84]. In general, DNA methylation in regulatory regions (e.g., promoters) reduces gene expression and represents an important mechanism for tissue-specific gene silencing [89]. In contrast, demethylation leads to gene activation [88]. When methylation occurs in a gene promoter, the methyl group can interfere with transcription factor binding. Alternatively, gene silencing may occur through targeting of DNA-binding proteins. MeCP-1 and MeCP-2 (methyl cytosine binding proteins 1 and 2) recognize methylated DNA and recruit corepressors and histone modifying enzymes such as histone deacetylases and histone methyltransferases to methylated genes, precipitating an inactive chromatin configuration [88]. Important to this proposal, key genes that regulate HPA function (GR, CRH, POMC, and 11*β*-HSD-2) have been shown to be epigenetically regulated [82, 89–93]. Altered gene promoter methylation has been identified in human diseases [90]. More recently, altered hippocampal GR promoter methylation patterns has been demonstrated in human suicide subjects [94]. Maternal stress/anxiety and dietary protein restriction during pregnancy [92, 93, 95–98] and altered levels of maternal care [82, 85, 99] can leave permanent epigenetic marks in the genome and result in stable life-long changes in gene expression in offspring. Szyf and Meaney have shown that increased maternal care in rats leads to demethylation and increased histone acetylation of the hippocampal *GR* promoter, which is maintained throughout life [82]. Importantly, this effect is gene-promoter specific; other nonhippocampal *GR* promoters are not affected. Demethylation was specific to the NGFI-A binding site in the *GR* promoter, resulting in increased NGFI-A binding and increased *GR *expression. This leads to increased glucocorticoid feedback and a decrease in HPA activity [82]. The reduced promoter methylation could be reversed in adulthood by central infusion of L-methionine (a methyl donor) [86]. In other studies, protein restriction during rat pregnancy has been shown to cause reduced methylation of specific hepatic gene promoters, including the *GR,* and a resultant increase in gene transcription in F_1_ offspring. Again, this demethylation is gene-specific and can be prevented by folate (a methyl donor) supplementation during pregnancy [97]. Very recent rat and human studies have shown that maternal stress/anxiety during pregnancy leads to altered methylation of the hypothalamic *CRH *promoter (rat offspring) and *GR* promoter in umbilical cord blood mononuclear cells (humans) [92, 93]. However, of most importance, glucocorticoids have been shown to cause permanent demethylation of specific fetal hepatic gene promoters in late gestation [100]. This demethylation results in enhanced transcription factor binding, and this is maintained following glucocorticoid withdrawal indicating stability of the effect [100]. One route by which sGC may modulate methylation status is through a reduction in folate availability. In this regard, Cushing's patients (with elevated plasma cortisol) exhibit hyperhomocysteinemia [101]. Hyperhomocysteinemia inhibits the activity of DNA methyltransferases and induces hypomethylation [102]. There is extremely strong evidence emerging indicating that fetal GC exposure, be it endogenous or exogenous in the form of ACS, has profound influences on the fetal epigenome.

### 8.2. Transgenerational Effects

Recent studies have begun to show that the effect of ACS exposure may not be restricted to the immediate offspring of the pregnancy at risk but may affect subsequent generations; this is known as “transgenerational effects”. These effects have been observed in the rat model where the offspring of an F_1_ progeny whose mothers were treated with dexamethasone in the final week of pregnancy were mated with males from the same prenatal treatment. Both the F_1_ and F_2_ generation offspring exhibited decreased weight at birth compared to controls and also demonstrated abnormal endocrine responses to glucose challenges [103]. The programming effects were transmitted by either maternal or paternal lines implying an epigenetic mechanism. Further studies are needed to elucidate these mechanisms and how this will translate into the clinical realm.

## 9. The Need for Focused Research on Long-Term Effects of ACS

Pregnant women at risk of preterm birth continue to be a major clinical obstetrical issue. A single course of ACS remains the standard of care in this clinical setting to optimize fetal lung maturity. Because of concerns of fetal growth alterations and potential neurodevelopmental impairments, systematic repetitive administration of ACS has not been adopted as standard care. However, because of the short-term benefits of reducing neonatal morbidity, the more recent Cochrane Review supports the use of this approach in identified high-risk women [42]. In addition, the concept of “rescue” ACS has emerged in which ACS is given again only when delivery has again become likely [104]. Recent data from two randomized trials demonstrate benefits in reducing acute neonatal pulmonary morbidity without noticeable effects on fetal growth [105, 106].

The long-term effects of antenatal sGC exposure on HPA axis activity and neurological function is well documented in animal studies, and it appears that sGC, regardless of dose exposure, is capable of affecting fetal HPA axis development causing permanent changes in the HPA axis that persists through life and is manifested by chronic illness and behavioral changes. The previous long-term studies of ACS have focused only on major neurodevelopmental difficulties (cerebral palsy, blindness, deafness, and cognition) to the age of 2 years. However, the more recent animal studies suggest that the long-term effects of any exposure to sGC are more related to behaviour and the cardiometabolic factors contributing to chronic mental and physical illnesses, none of which have been adequately assessed in the majority of the long-term human follow-up studies. In retrospective studies, there is some suggestion of the behaviour changes seen with repeated exposure to sGC [46]. If early exposure affects hippocampal structure and alters function as the myriad of studies in animal models suggest, then there is likely to be an explosion of cognitive challenges, behavior disorders, and, perhaps, the risk of psychological disorders [107]. Similarly, if there is significant alteration in the HPA axis, then a generation of children and perhaps beyond are prone to develop the chronic illnesses of adulthood, such as heart disease, hypertension, type 2 diabetes.

The use of ACS, single or multiple courses, in the obstetric management of women at risk for preterm birth is likely not to diminish. Further studies are required on how to optimize the use of ACS in those women who remain undelivered 7 to 10 days after receiving an initial course of ACS. In addition, it is critical to determine ACS role, regardless of dosage, in “fetal programming” and its potential impact on a generation of children as it relates to behaviour, learning skills, and the potential for chronic illness. If the impact can be identified, appropriate measures can be implemented to minimize its effect.
